# Assessment and Diagnosis of HIV-Associated Dementia

**DOI:** 10.3390/v15020378

**Published:** 2023-01-28

**Authors:** Amalia Cornea, Irina Lata, Mihaela Simu, Elena Cecilia Rosca

**Affiliations:** 1 Department of Neurology, Victor Babes University of Medicine and Pharmacy Timisoara, Eftimie Murgu Sq. No. 2, 300041 Timisoara, Romania; 2 Department of Neurology, Clinical Emergency County Hospital Timisoara, Bd. Iosif Bulbuca No. 10, 300736 Timisoara, Romania

**Keywords:** HIV, AIDS, dementia, cognitive impairment

## Abstract

The modern combined antiretroviral treatment (cART) for human immunodeficiency virus (HIV) infection has substantially lowered the incidence of HIV-associated dementia (HAD). The dominant clinical features include deficits in cognitive processing speed, concentration, attention, and memory. As people living with HIV become older, with high rates of comorbidities and concomitant treatments, the prevalence and complexity of cognitive impairment are expected to increase. Currently, the management of HAD and milder forms of HAND is grounded on the best clinical practice, as there is no specific, evidence-based, proven intervention for managing cognitive impairment. The present article acknowledges the multifactorial nature of the cognitive impairments found in HIV patients, outlining the current concepts in the field of HAD. Major areas of interest include neuropsychological testing and neuroimaging to evaluate CNS status, focusing on greater reliability in the exclusion of associated diseases and allowing for earlier diagnosis. Additionally, we considered the evidence for neurological involvement in severe acute respiratory syndrome coronavirus 2 (SARS-CoV-2) infection and the impact of the coronavirus (COVID-19) pandemic, with wider consequences to population health than can be attributed to the virus itself. The indirect effects of COVID-19, including the increased adoption of telehealth, decreased access to community resources, and social isolation, represent a significant health burden, disproportionately affecting older adults with dementia who have limited social networks and increased functional dependence on the community and health system. This synopsis reviews these aspects in greater detail, identifying key gaps and opportunities for researchers and clinicians; we provide an overview of the current concepts in the field of HAD, with suggestions for diagnosing and managing this important neurological complication, which is intended to be applicable across diverse populations, in line with clinical observations, and closely representative of HIV brain pathology.

## 1. Introduction

Globally, about 38.4 million people are infected with the human immunodeficiency virus (HIV) [1], accounting for 0.7% of the population aged between 15 and 49 years. However, the epidemiological data varies considerably between countries and regions. The highest prevalence is estimated in the African Region, with approximately 1 in every 25 adults (3.4%) being HIV-positive, which accounts for over two-thirds of people living with HIV (PLWH) worldwide. Therefore, millions of individuals have or are at risk of developing cognitive impairment [1].

Despite recent advances in managing and treating infected individuals, HIV-associated neurocognitive disorders (HAND) were reported in between 30 and 60% of adults [2,3,4,5,6,7]. Epidemiological studies report that HIV-associated dementia (HAD) is rare nowadays (2–4%) [7], and most PLWH present mild forms of cognitive dysfunction, including asymptomatic neurocognitive impairment (ANI) and mild neurocognitive disorder (MND) [4,8,9]. A systematic review of the literature reported a pooled prevalence rate for HAD, according to the Frascati criteria of 5% (95% CI 3.5–6.8) [2]. The authors found a global prevalence of HAND in 42.6%, with 23.5% (95% CI 20.3–26.8) of cases presenting ANI and 13.3% (95% CI 10.6–16.3) of individuals being diagnosed with MND [10]. Another recent meta-analysis reported similar results, with an incidence of HAD at 2.1% (95% CI 1.2–3.7%) [3]; in contrast, the estimated prevalence of HAND, ANI, and MND were 44.9% (95% CI 37.4–52.7%), 26.2% (95% CI 20.7–32.7%), and 8.5% (95% CI 5.6–12.7%), respectively [3]. 

The prevalence of HAD and MND decreased with income level, current CD4 count, the proportion of PLWH on cART, and age. By contrast, the prevalence of ANI increased with age. However, this data could be explained by the fact that the pooled proportion of cART and current CD4 count increased with age [10]. 

A higher HAND prevalence, including HAD, was found in Latin America, the Caribbean region, and PLWH with a low nadir CD4 count (<200 cells/mm^3^). The prevalence of HAND, in general, did not vary by the percentage of individuals receiving combined antiretroviral treatment (cART), the current CD4 count, or the proportion of patients with hepatitis C virus (HCV) coinfection.

The introduction of cART in 1996 was a landmark in HIV history, as effective this antiretroviral treatment decreased the severity of neurological impairments. Although the frequency of HAND did not decrease, with an unchanged prevalence of cognitive impairment in PLWH with systemic viral suppression, dementia is rare, while the milder, non-demented forms of HAND account for most cases [4]. 

## 2. A Brief History and Current Diagnostic Nomenclature of HIV-Associated Neurocognitive Disorder

In 1986, Navia et al. delineated a triad of clinical symptoms comprising cognitive impairment, motor dysfunction, and behavioral changes in the context of acquired immune deficiency syndrome (AIDS), which were later termed the AIDS dementia complex (ADC) [5]. The authors reported that, among 70 autopsied patients with HIV, 46 presented progressive dementia and were often accompanied by motor and behavioral symptoms. The majority of ADC cases (63%) had a previous diagnosis of AIDS, but 37% of the patient’s developed ADC before any clinical evidence of AIDS [5]. Furthermore, several individuals displayed only some, but not all, the symptoms necessary for the ADC diagnosis. These findings indicated that the label ADC was inaccurate. 

The first comprehensive investigation of neurocognitive deficits in PLWH was published in 1987, providing robust evidence of objective cognitive impairments across all stages of the HIV disease, from asymptomatic HIV infection to AIDS [6]. The patients presented deficits in several cognitive domains, including executive functions, episodic memory, and cognitive processing speed [6]. Consequently, in 1990, the World Health Organization (WHO) advocated a new diagnostic term, HAD, to substitute ADC [7]. The American Academy of Neurology (AAN) also endorsed this new term, with a distinction between HIV-1-associated minor cognitive/motor disorder (MCMD) and HAD [8]. For a HAD diagnosis, a patient needed to present an acquired cognitive impairment in at least two neurocognitive domains that affected the individual’s work or activities of daily living (ADLs) and abnormalities in motor abilities or specific neuropsychiatric or psychosocial functions (e.g., motivation, lability, and social behavior). Compared with HAD, MCMD included patients with a less severe neurocognitive impairment who still met the criteria for HAD but presented impairment in at least two cognitive or behavioral areas, causing a decline in ADLs [8]. In this classification, MCMD and HAD may denote cognitive dysfunctions along a continuum, but they may also exist as separate entities [8]. 

Further research on the neuropsychological aspects of HIV infection, demonstrating in PLWH mild cognitive deficits that did not interfere with ADLs, along with the changing epidemiology of HIV infection, has led to the need to update and reorganize the HAND diagnostic criteria [9]. 

In 2007, the nosology of HAND was reconsidered and amended with criteria allowing for three possible diagnoses: ANI, MND, and HAD [9]. For a diagnosis of ANI, the patient’s neuropsychological test scores should be at least one standard deviation (SD) below the normative data in at least two cognitive domains with unimpaired daily functioning. For MND, PLWH should demonstrate comparable neuropsychological test results but with impaired daily functioning. For a diagnosis of HAD, the patient must present severe deficits in at least two domains, typically two SDs below normative data and more severe impairment in ADL [9]. Although these criteria were intended for use in research, the terminology has become widely used to refer to the clinical disease burden [10]. 

While there is a general agreement that PLWH with advanced disease presents with deficits in several cognitive domains, there is a debate on evidence regarding whether deficits emerge in asymptomatic individuals. In recent years, researchers have challenged the validity of these criteria [10,11,12], questioning the clinical applicability of ANI and the validity of the cognitive assessment in characterizing HAND. Some scientists consider that testing for ANI is not justified, as there is no highly specific or sensitive screening tool that can be used in all clinical settings. Additionally, there has yet to be an agreement on the therapeutic strategy for asymptomatic PLWH. A false positive screening test might lead to unnecessary or costly diagnostic procedures and cause additional distress to some individuals [10,13]. Additionally, some longitudinal studies reported that ANI progresses only rarely to a symptomatic status [14], and several researchers found no association between the use of cART with a high central nervous system (CNS) effectiveness and the neurocognitive status [15,16,17].

Conversely, some researchers advocate screening for ANI, as PLWH with ANI have poor adherence to treatment with high rates of unemployment [18]. Additionally, ANI was associated with a higher risk of progressive cognitive dysfunction [14]. In some studies, using cART with high CNS effectiveness reported improved cognitive status [19]. Changing cART according to the estimated CNS effectiveness decreased HIV RNA levels in the cerebrospinal fluid (CSF), improving neurocognitive performance [20]. Moreover, some ART was documented to present neurotoxic consequences [21]. Additionally, recent studies reported grey and white matter abnormalities in individuals with ANI [22] and abnormal plasma biomarkers (e.g., nadir CD4 count, neurofilament light chains, neopterin) [23].

## 3. Clinical Manifestations and Cognitive Profile of Dementia

As a consequence of HIV infection within the CNS, the advanced stages of the disease are associated with cognitive dysfunction [24]. At the advent of the HIV epidemic, many PLWHs developed severe neurological manifestations in the final months of the illness, with progressive subcortical dementia characterized by motor and cognitive slowing [25]. The clinical syndrome of cognitive, behavioral, and motor symptoms was labeled ADC. However, after the introduction of cART, a predominantly cortical, more so than the subcortical pattern, was constantly documented [26]. 

HAD preferentially affects the fronto-striato-thalamo-cortical circuits. In the early stages of HIV infection, the neurological examination is frequently normal. Sometimes, the patients may present generalized hyperreflexia and rapid eye and limb movement impairments. As the disease advances, their cognitive status deteriorates, and neurological examination may detect hypertonia, particularly in the lower limbs, tremors, clonuses, and frontal release signs. Severe movement disorders, including parkinsonism, tremors, chorea, ballism, myoclonus, dystonia, and paroxysmal dyskinesias, are infrequent in HIV infection but can occasionally appear in patients with HAD [27,28]. In addition, extrapyramidal signs may be present in PLWH, particularly in advanced stages [29].

Furthermore, bradykinesia (i.e., slowed movement) and bradyphrenia (i.e., slowed information processing) are widely identified as one of the most prominent traits of neuroAIDS, with motor slowing is a required feature for the HAD diagnosis in the earlier diagnostic criteria [8]. Motor slowing can manifest as decreased gait velocity, finger tapping, and manual dexterity impairments [30]. The cognitive and motor slowing are heightened by processing demands, such as circumstances requiring divided attention [31]. In the end stages, the patient is usually incontinent and mute [32]. 

Neurocognitive dysfunction comprises, in particular, mental slowing, attention, and memory deficits. Although dementia incidence decreased with the introduction of cART, PLWH with treatment and long-term infection present now mild neurocognitive symptoms. Additionally, specific demographic characteristics and risk factors for cognitive impairment have shifted to increased age and cardiovascular risk factors. Hence, the HAND phenotype is extended by broadening the neuropsychological profile [30,33]. Patients with dementia demonstrate a subcortical profile of cognitive impairment, the core deficits including mental slowness, attention and memory deficits, and executive dysfunction [30]. A common cognitive abnormality is a decreased information processing speed [30,33,34]. As mental speed promotes most cognitive and motor functions, some authors consider it a fundamental deficit, causing dysfunctions in other cognitive domains [35]. 

Attention and working memory are likewise affected. The ability to generate memory for temporary processing and storing information depends on attention functions, so these neurocognitive functions are interconnected. Accordingly, these deficits overlap [30,34]. Episodic memory impairments are often reported in HIV-positive individuals and, together with psychomotor slowing, are regarded among the most sensitive indicators of HAND, including HAD [36]. In advanced disease, episodic memory impairment is moderate to severe [37]. In addition, learning new information and prospective episodic memory deficits are also present, with the patient’s ability to execute a subsequent intention or “remembering to remember” is impaired [34].

In PLWH, particularly in advanced disease stages, the executive functions are affected, with poor reasoning, planning, problem-solving, and shifting between tasks [34,37,38]; this substantially impacts everyday functioning [39].

The most frequent language problem is impaired fluency, which could also originate from mental slowness or executive dysfunction. More rarely, patients with HAND may present impaired sensory-perceptual functions, with difficulties in interpreting and integrating visual, auditory, or sensorial stimuli [34]. 

Visuospatial cognition was less investigated in PLWH. Patients may present impairments in the ability to detect, understand, manipulate, and integrate visual stimuli in the context of their surroundings. However, subtle deficits are invariably present, even without primary visual dysfunction [30].

## 4. Diagnosis of HAD

Whereas ANI and MND are assumed to be a lesser form of dementia on a continuum of neurocognitive impairment, when representing neuronal dysfunction, HAD is considered to represent neuronal death. 

Although the Frascati criteria were formulated for research purposes, the classification has been regularly adopted into clinical practice [9]. 

By definition, HAD represents a moderate to severe acquired cognitive impairment that was documented by a score of at least 2 SD below the demographically corrected normative data in at least two different cognitive areas; the deficit is commonly present in multiple domains, particularly in learning, information processing speed, attention, and concentration. In addition, in HAD, cognitive impairment causes significant interference with day-to-day functioning. Additionally, the pattern of cognitive impairment in HAD does not meet the criteria for delirium, and comorbid conditions do not satisfactorily explain dementia syndrome [9].

Standard neurological evaluation and simple bedside testing may be used if extensive neuropsychological testing is unavailable. 

By contrast, MND is considered an acquired mild-to-moderate cognitive impairment, which is documented by a score of at least 1 SD below the demographically corrected normative data on tests of at least two different cognitive domains; the cognitive impairment interferes mildly with daily activities [9]. 

The ANI is diagnosed in PLWH with a performance of at least 1 SD below the normative scores in at least two cognitive domains (attention, information processing, language, abstraction, executive functions, complex perceptual-motor skills, learning and recall memory, simple motor skills, and sensory-perceptual abilities); these criteria specify that at least five cognitive domains need to be evaluated. Most importantly, cognitive impairment does not alter everyday functioning [9].

Similar to HAD, in MND and ANI, the cognitive impairment does not meet the criteria for delirium and is not fully explained by comorbidities.

Worldwide, there is a consensus between guidelines concerning the diagnosis of HAND [40]. However, although referring to cognitive impairments, no guideline offers an explicit definition for HAD or the milder forms of HAND [9]. Some guidelines have a specific section regarding cognitive impairment, advocating a thorough evaluation that includes a rigorous anamnesis and examination, depression screening, neuropsychological assessment, magnetic resonance imaging (MRI) of the brain, and lumbar puncture [41,42,43]. They recommend a complex neuropsychological assessment testing several cognitive domains and endorse several preferred tests for each cognitive domain, but consensus on specific tests needs further refining. The tests should have appropriate normative data that is validated in the language and culture of the specific population [42]. 

Nevertheless, most of these tests are available in a limited number of centers, and their administration requires highly qualified staff [42]. Consequently, it would be more convenient to use short, easily accessible screening tests with high psychometric properties that can be administered by clinical personnel across diverse clinical settings. However, several guidelines do have explicit recommendations on screening for cognitive impairment, and substantial variation reflects the uncertainties in this field [40]. 

The European AIDS Clinical Society [41] advocates for the screening of all individuals without confounding conditions (such as severe psychiatric pathology, abuse of psychotropic drugs or alcohol, the use of anticholinergic medication with a high burden score for cognitive dysfunction, concomitant CNS opportunistic infections or other neurological pathology, sequels of brain disorders, pre-treatment neurocognitive disease, or other neurological diseases) at the time of HIV diagnosis, before staring cART and subsequently as indicated based on symptoms. The screening method includes three questions: “Do you experience frequent memory loss?”, “Do you feel slower when reasoning, planning activities, or solving problems?” and “Do you have difficulties paying attention?”. A positive answer to at least one question suggests the possibility of cognitive impairment requiring additional evaluations [41]. The Mind Exchange Program proposes screening PLWH in the first six months of HIV diagnosis, before cART initiation, every 6 to 12 months if they present a high risk, every 12 to 24 months if they have a low risk, and promptly if there is any clinical decline [42]. The risk factors and comorbidities for HAND and implicitly HAD and/or non-HIV-related cognitive impairment include disease factors (i.e., low nadir CD4 cell count, high plasma or CSF HIV RNA, low current CD4, history of HIV-related CNS diseases, longer HIV duration), treatment factors (i.e., low cART adherence, episodes of cART interruption, non-optimal cART regimen, short cART duration), and comorbidities (i.e., positive HCV serostatus with high HCV RNA, history of acute cardiovascular event and presence of cardiovascular risk factors, anemia, and thrombocytopenia). Additionally, demographic factors (i.e., older age, low educational level, ethnicity, and sex) and other neurological and psychiatric factors (i.e., depression, anxiety, post-traumatic stress disorder, psychosis, bipolar disorder, alcohol or illicit drug abuse, syphilis or systemic infection, Alzheimer’s disease (AD), cerebrovascular diseases, traumatic brain injury, vitamin, or hormone deficiency) that may present a high risk for developing cognitive impairment. Likewise, some aspects of cART medication, such as low CNS penetration efficiency, and neurotoxicity, may increase the risk for HAND [42].

The Mind Exchange Working Group advises the use of specific screening tools depending on the availability of a clinician with suitable training for administering and interpreting each test. Opting for one specific screener depends on whether the assessor intends to detect HAD or milder HAND forms. The time, financial costs, and population characteristics also influence the choice [42]. As neuropsychological resources are restricted in many clinics, a probable clinical diagnosis of HAND, including HAD, could be established based on questionnaires, screening tests, functional assessments, and short neuropsychological assessments. If the screening is positive, PLWH could be referred to for a complete neuropsychological assessment [42]. The screening tests endorsed by the Mind Exchange Working Group include HIV Dementia Scale (HDS) and the International HIV Dementia Scale (IHDS) [42]. 

The British HIV Association advises that screening for cognitive dysfunction should be available for all individuals within three months of receiving the HIV diagnosis. Additionally, all PLWHs should undergo periodic screening after events that are likely to trigger or exacerbate cognitive impairment; otherwise, screening should be conducted annually [44]. Likewise, the Infectious Diseases Society of America has comparable recommendations [45]. The WHO endorses routine screening and management of mental health disorders for PLWH in key populations to optimize health outcomes and improve cART adherence. However, the methods and frequency of screening are not mentioned [46]. The Italian Society for Infectious and Tropical Diseases advises screening all PLWH with cognitive complaints. Among the proposed tests, they recommend Montreal Cognitive Assessment (MoCA) and Cogstate [43]. 

Similar to other cognitive disorders, HAD diagnosis cannot be made in the setting of an acute illness. In stable PLWH with neurocognitive symptoms, several screening tests are accessible and easy to administer in outpatient settings. If screening detects cognitive impairment, the diagnosis should be confirmed by a comprehensive neuropsychologic assessment that investigates multiple cognitive domains. These include verbal fluency, executive functions, information processing speed, attention, working memory, verbal and visual learning alongside memory, motor skills, and ADL. 

In addition to neuropsychological evaluation, a neurological examination, brain MRI, CSF analysis, and electrophysiological studies (EEG, somatosensory evoked potentials) are warranted to exclude other causes of cognitive impairment. However, HAD is a clinical diagnosis, and no paraclinical finding in isolation certifies the HAD diagnosis. 

In patients on cART, the investigation of possible CSF viral escape and evidence for genotypic drug resistance (GDR) in a paired CSF and plasma sample further refines the management of cognitive impairment [41]. After the HAD diagnosis is made and any additional causes of cognitive impairment are excluded (e.g., cART toxicity), specific treatment and care steps should be taken [41]. 

### 4.1. Screening Tests for HAD

To date, a considerable number of studies have investigated the diagnostic accuracy of many screening tests in detecting HAD and the milder forms of HAND. A systematic review performed in 2013 found 36 studies investigating the diagnostic accuracy of 40 different screening tests or short neuropsychological batteries [47]. In the next five years, 23 further studies were published, providing data on 18 screening tools, including several that were novel computer-based or mobile device-based [48].

Interestingly, most of the literature on cognitive impairment focuses on HAND, minimizing the data on HAD. For example, in a systematic review of the MoCA test in PLWH, among eight included studies, there was insufficient data to perform a meta-analysis for the HAD subgroup [49]. In another systematic review on IHDS, among 15 studies on HAND, only six studies provided data on the use of IHDS in detecting HAD [50].

Furthermore, only a few screening tools have been developed and validated specifically for PLWH. The HDS, and its derivative form, IHDS [51,52], are most frequently used among them. Although expert guidelines endorse them, both tests have a relatively low sensitivity for the mild cognitive symptoms predominating in the cART era [53]. 

The HDS was primarily developed for use in outpatient settings to identify HAD. It investigates motor speed, concentration, and memory and is fast to administer (between 3 to 5 min), easy to score, and interpret. However, its administration is limited because it requires a trained examiner to evaluate the antisaccadic eye movement. Additionally, the alphabet writing and cube copying subtests may be challenging for PLWH with a non-Western educational background [42]. For HDS, the pooled diagnostic odds ratio (DOR) was reported to be 7.52 (95% confidence interval 3.75–15.11), with a sensitivity of 68.1% and a specificity of 91.2% in detecting HAD [54]. The diagnostic accuracy of HDS improves by increasing the threshold and using demographically adjusted T-scores, but only for detecting HAD, not the milder forms of HAND [55]. 

The IHDS was developed for use in international settings, with limited resources for HIV individuals with various cultural, linguistic, and educational backgrounds [51]. It does not require specific training, is easy to integrate into clinical practice, and evaluates memory, motor speed, and psychomotor functions [36]. It requires only 2–3 min to administer; also, its use demands only a stopwatch and is easy to score. A systematic review and meta-analysis found that for detecting HAD, the optimal cutoff point was 9.72, with a sensitivity of 79.4% (95% CI 46.8–94.4%], a specificity of 65.4% (95% CI 33.5–87.6%), and an AUC = 0.777 (CI: [0.597–0.893] [0.554–0.91]) [50]. Following the pooled estimation of the accuracy for detecting cognitive impairment in different subgroups (including HAND, symptomatic HAND, and HAD), the authors proposed as a common threshold a score of 10. In this case, IHDS presents a specificity of 8.56% (95% CI 57.0–96.4%) and a sensibility of 58.1% (95% CI 27.0–83.8%) in detecting HAD [50].

Among general cognitive screeners, the mini-mental state examination (MMSE) has the advantage of being widely used in clinical practice for detecting cognitive disorders of various causes, and clinicians are familiar with it. Although it is the first-choice screening test in many cases, several studies have demonstrated that MMSE has low diagnostic accuracy in PLWH [53,56,57]. 

The MoCA, a short bedside test developed for detecting mild cognitive impairment, was documented to present high sensitivity and specificity in older adults [58]. Additionally, it is used in a wide range of specific populations (i.e., mild cognitive impairment, AD [59], Parkinson’s disease [60], Huntington’s disease [61], multiple sclerosis [62], schizophrenia [63], and cerebrovascular disease [64]). It has the advantage of assessing cognitive functions that are commonly affected in PLWH, including short-term memory, attention, working memory, and frontal-executive abilities. However, recent research found that a lower threshold than the original cutoff of 26 was more advantageous for HAND screening, lowering false positive rates and improving diagnostic accuracy [49]. For HAND, the best balance between true positive and false positive test results was obtained with a threshold of 23 (sensitivity of 44% and specificity of 79%) [49]. Nonetheless, data from the included studies did not allow meta-analysis for the specific diagnosis of HAD. 

Other screening tools that may be useful for diagnosing HAD include the total recall subtest of the Hopkins verbal learning test-revised, grooved pegboard test [36], and Executive Interview [65]. Their benefits and limitations are presented in the report of the Mind Exchange Program [42]. Additionally, further details on other tests are outlined elsewhere [48].

Although the traditional paper-and-pencil tests demonstrate undoubted value, they also have drawbacks. Human errors may occur in their administration and scoring or when the assessor manually enters the data into electronic medical records. Consequently, several computerized tests such as Cogstate [66] and Neuroscreen [67,68] have been developed, with promising results. For example, CogState presented a sensitivity of 100% and a specificity of 98% in detecting MND and HAD [69]. However, for HAND, including milder forms of cognitive impairment, the sensitivity and specificity decreased from 76% to 71%, respectively [69]. While computer or mobile-based screening tools have certain benefits, they are more expensive. Further research is needed on their diagnostic accuracy and the feasibility of incorporating them into clinical settings. 

Recently, during the Coronavirus disease 2019 (COVID-19) pandemic, there was an increased practice of teleneuropsychology (e.g., video teleconferencing and other platforms for the delivery of neuropsychological services). This type of neurocognitive assessment provides many benefits, mainly as it allows neuropsychological and other clinical services to continue remotely during shutdowns or situations that limit person-to-person contact. When used carefully with a clear understanding of their limitations, telephone adaptations provide an opportunity to continue monitoring patients and promote equity. In PLWH, telephone screeners and face-to-face evaluations were compared to examine whether test performances differed between administration types and the levels of pre-pandemic cognitive performance. Over 90% of PLWH in the study gave positive feedback about the telephone encounter. Telephone and face-to-face test scores did not differ significantly for category fluency, letter fluency, and verbal learning measures [70]. The results support the telephonic adaptation of selected face-to-face measures, with verbal fluency tasks showing the most robust equivalency [70].

Furthermore, recent systematic reviews found that for a diagnosis of all-cause dementia, the sensitivity of telehealth assessment ranged from 80% to 100% and specificity from 80% to 100%. Nonetheless, the authors assessed the findings as very low-certainty evidence due to imprecision, inconsistency between studies, and the risk of bias [71]. Additionally, these estimates were considered imprecise due to small sample sizes and between-study heterogeneity. They may apply specifically to telehealth models, which incorporate a large amount of face-to-face contact with healthcare providers other than the doctor responsible for making the diagnosis [71].

Another potential barrier to the use of screening tests is related to the age of PLWH, as there are only a handful of studies on children and individuals older than 50 years [48,72]. Additionally, when using a screening test, the assessor must consider its psychometric validity in a specific population and its cultural and demographic suitability. For example, some drawing sub-tests (e.g., cube drawing) are not well designed for PLWH in South Africa and could incorrectly indicate cognitive impairment [73].

An additional challenge includes the lack of normative data for specific populations. Many countries with a high HIV burden do not have formally validated and normed neurocognitive tests; this makes it impossible to understand how the individual’s performance compares to the general population.

### 4.2. Functional Assessment

Although the Frascati criteria are widely used, and in order to fully diagnose dementia and the milder forms of cognitive impairment, a functional assessment is required, research in this area remains scarce. Only a few functional assessment tools are available for PLWH across countries and contexts [48]. 

The most frequent approach is a single self-report ADL questionnaire, which is typically dichotomized as “dependent” when difficulties are apparent in two or more domains (i.e., finances, shopping, and planning social activities) [74]. Other tests include performance-based measures of functional ability, such as medication management or vocational tasks to improve sensitivity [75]. Multimodal approaches, including the number of impaired ADL measures, performance-based tasks, estimates of cognitive symptoms, and vocational status, are seldom used [76,77].

The criteria and specific measures that are used to classify functional impairments can significantly influence the diagnosis of HAD. The levels of daily functioning vary considerably within and across populations, depending on socioeconomic and cultural factors. These factors need to be considered when in local and international settings. For example, across societies, gender roles may vary. Additionally, some tools often include inappropriate items for specific contexts, such as the ability to handle finances via check writing or operating bank accounts. Many PLWHs from certain regions do not have bank accounts or do not use checks. Therefore, the type of daily activities should be adapted to the context in which the test is intended for use [48].

### 4.3. Extensive Neuropsychological Testing

The reference standard for diagnosing HAND is a comprehensive neuropsychological battery that investigates several cognitive domains, including attention, working memory, language, abstraction, executive functions, information processing speed, learning and recall, and motor and sensory-perceptual skills. HAD diagnosis requires the performance of 2 SD below the normative data, in at least two cognitive domains, with a significant impairment in daily functioning [42]. 

Despite a comprehensive investigation of cognitive functions, this approach also has several limitations. First, it is time-consuming, requires trained personnel, and is more expensive and problematic to implement in clinical practice. Second, in normal populations, without HIV infection, 15–22% of individuals may present false-positive test results for HAND diagnosis [78]. Nonetheless, only 2.2% or 2.3% of the population perform worse than 2 SD below the mean [11,78]. Using the HAD criteria (Z scores ≤ 2 SD), false-positive rates range from 0 to 8%, with a threshold Z score of ≤2 SD being acceptable for HAD [78]. However, this may have important implications in research, as even low false-positive rates may present significant effects on power when the true prevalence of the disease is low, as for HAD. For example, a false-positive frequency of 1% may significantly affect the effective sample size, reducing an actual sample size of 100 to an effective sample size of 55. This reduction is even more important for larger sample sizes, as an actual sample size of 1000 would be reduced to 186 [78].

Extensive cognitive evaluations with several tests imply several comparisons that yield high false-positive rates compared to a single test; the likelihood of an abnormal score increases with the number of tests for each cognitive domain and the number of investigated domains. These false-positive results are due to the methods intended to increase the sensitivity of neuropsychological batteries in detecting mild forms of cognitive impairment. Additionally, high threshold scores (Z scores with a cutoff of 1 SD), which are used to detect mild HAND forms, can induce an important overlap between critical portions of test-score distributions in PLWH with and without cognitive impairments [11,78]. Consequently, the cost of a battery with high sensitivity would be a reduction in specificity. Accordingly, it is recommended that individual tests should be grouped into a maximum of five cognitive domains [78].

## 5. The Role of Comorbidities in HAD

The presence of several confounding factors can hinder HAD diagnosis. First, the diagnostic criteria recommend assessing day-to-day functioning. However, the methods to obtain evidence of ADL impairments need further refinement. Additionally, a decreased performance on cognitive testing may be due to socioeconomic factors, low educational levels, and comorbidities, as they may interfere with neuropsychological testing results [10].

The diagnosis of HAD implies that cognitive dysfunction is due to the direct effects of HIV infection on the CNS. Nevertheless, in daily practice, there are three categories of patients with cognitive impairment: individuals with HAD caused solely by HIV, PLWH with cognitive dysfunction resulting from a combination of factors (i.e., HIV and comorbidities), and individuals in which HIV pathology may not contribute at all to the decreased performance of neuropsychological testing.

PLWH may present several comorbidities that significantly impact CNS functions. For example, hypertension, dyslipidemia, diabetes, and obesity are well-established risk factors for cerebrovascular disease and cognitive impairment [79]. Studies report a 6.2-fold higher risk of cognitive impairment in PLWH with associated cardiovascular risk factors [80]. Additionally, hyperglycemia and atherosclerotic disease in older patients are associated with lower psychomotor speed performance [81]. 

Cerebrovascular diseases, particularly associated with atherosclerosis, were a rare complication before the cART era. However, after the introduction of cART, a substantial increase in the proportion of PLWH over the age of 65 was noted, with cerebrovascular diseases becoming more prevalent with changes in the etiology of treated, aging individuals [82]. The pattern of illnesses closely resembles that of the older general population. However, in individuals with HIV infection, there are also significant differences regarding disease etiology, progression, treatment, and risk stratification [82], as cerebrovascular diseases may result from both traditional risk factors (i.e., smoking) and the effects of HIV and cART on the endothelial function [83]. Cerebrovascular diseases are likely to contribute to a significant burden of HAND, including HAD in an older cohort, possibly with an altered phenotype. Additionally, an increase in the prevalence of vascular dementia can be expected. 

Aging is another risk factor for dementia. As the life expectancy of PLWH increases, they might also develop multiple geriatric syndromes in the context of aging and multi-morbidity [84]. Studies demonstrate that individuals over 50 with HIV present a risk of developing mild cognitive impairment that is 7-fold higher than HIV-negative age-matched controls. However, although HIV infection might accelerate cognitive aging and dementia [85], in daily practice, it is challenging to anticipate which individuals with ANI or MND could progress to dementia or what type of dementia they would develop [86]. 

AD shares several pathological features with HAD. For example, Apolipoprotein E epsilon 4 (APOE-ε4) is an essential factor in the pathogeny of AD. In PLWH, at least one APOE-4 allele was associated with brain atrophy and decreased cognitive functioning in several domains (i.e., attention and working memory, executive functions, and verbal fluency) [87,88]. In HAND patients, particularly those above 65 years, the progression of symptoms and lack of impairment reversal may imply the overlap of AD [89]. Although very difficult, it is essential to differentiate AD from HAND, particularly from a therapeutic point of view.

Another risk factor for cognitive dysfunction in PLWH is coinfection. Namely, coinfection with hepatitis C [90], a history of sexually transmitted diseases (e.g., syphilis, gonorrhea) [91], or cytomegalovirus [83] increases the risk of cognitive impairment. 

The severe acute respiratory syndrome coronavirus 2 (SARS-CoV-2) coinfection represents a significant risk factor for mortality in PLWH, especially if HAD is present. In the general population, among patients with dementia, the most frequent onset symptoms of COVID-19 were delirium, mainly in the hypoactive form, and a worsening of their functional status. This atypical clinical presentation may reduce early recognition of symptoms and hospitalization [92]. In addition, the presence of HIV infection, dementia, severe mental illness, cardiovascular disease, hypertension, chronic kidney disease, diabetes, COPD, and asthma is associated with increased severity and mortality [93]. 

Furthermore, failing to mitigate the negative effects of SARS-CoV-2 on daily life (e.g., isolation) and the health system is associated with preventable death among older adults with dementia [94]. For people with dementia diagnosed with SARS-CoV-2 infection, these disturbances may be associated not only with an increased risk of death due to SARS-CoV-2 infection but also with a high risk of death unrelated to COVID-19 [95]. 

In SARS-CoV-2 infection, there are five major categories of neurological involvement: encephalopathies (with behavioral disturbances, seizures), inflammatory CNS syndromes (e.g., meningoencephalitis, acute disseminated encephalomyelitis), strokes (both thrombotic and hemorrhagic), peripheral neurological disorders (e.g., Guillain-Barré syndrome, and CNS disorders, which do not fit into these categories [96,97]. The symptoms range in severity from mild (e.g., headache and loss of smell/taste) to severe (e.g., encephalitis) and may occur before or after other classical symptoms of COVID-19 [96].

Apart from the potential neurovirulence of SARS-CoV-2, the infection may lead to neurological and cognitive consequences via indirect routes that are linked to systemic infection and aggressive treatment. Patients with acute respiratory distress syndrome (ARDS) are at risk of cerebral hypoxemia [98]. Intubation and the use of mechanical ventilators are procedures with substantial risks. Additionally, the cytokine storms can lead to multiple organ damage, including renal and hepatic, liver, and cardiac dysfunction [99], all of which can adversely affect cognitive functioning. The SARS-CoV-2 may also cause CNS-related autoimmune disorders via molecular mimicry [100]. 

Assuming that SARS-CoV-2, similar to other human coronaviruses, can have a long-lasting presence in the CNS, its potential to provoke a chronic neuroinflammatory immune response that is similar to HIV-1 should be considered in the pathogenesis of cognitive deficits [96]. Furthermore, the long-term tracking of survivors should determine whether CNS exposure to SARS-CoV-2 increases the risk of later neurodegenerative diseases [96].

In addition, and similarly to HIV infection, COVID-19 is associated with a greater risk of psychiatric illness, which can exacerbate or complicate cognitive impairment. Considering the relatively increased fatality rate among individuals with dementia and SARS-CoV-2 infection, as well as the emotional and financial distress caused by the pandemic, psychiatric disorders such as post-traumatic stress disorder and depression should be considered as diagnoses and contributing factors to cognitive impairment [101]. Similarly, studies from the earlier SARS and Middle East respiratory syndrome (MERS) outbreaks reported high rates of post-traumatic stress disorder [102,103]. 

Finally, the cognitive assessment of PLWH with active symptoms of SARS-CoV-2 infection should be avoided, as even mild upper respiratory viral infections can have acute effects on cognitive functioning [104].

Psychiatric illness is a well-established factor affecting cognitive functions and likely aggravates the clinical picture of HAD [105,106]. The most frequent psychiatric comorbidity in PLWH is depression. In addition to mimicking cognitive impairment, it has a negative impact on cognitive functions [83]. A recent systematic review reported that the median rate for current depression was 24% in studies using self-report questionnaires or structured diagnostic interviews [107]. The lifetime prevalence of depression in PLWH was 42% [107]. The severity level was found to be “serious” in 80% of individuals [108]. The cognitive areas constantly associated with depression were information processing speed, executive functions, learning, memory, and motor function [107]. Depression was reported in between 0% and 31% of HAD patients (median 3%). By contrast, in MND, it ranged between 5% and 31% of individuals (median = 11%), and in ANI, it was demonstrated at a range from 10% to 55% (median = 30.5%). After excluding individuals with severe depression, a depression rate of 35% was reported in HAD patients, while in MND and ANI, it was 40% and 15%, respectively [109]. Studies not excluding PLWH with severe depression reported higher rates of depression among those with HAD (58%) [110]. Furthermore, PLWH with depression had a 1.4–3.1-fold increase in odds of developing MND or HAD compared to PLWH without depression [107].

Apathy may also have a negative impact on cognitive functions. Furthermore, neuropsychiatric conditions such as apathy are also manifestations of the disease, as acknowledged by the initial HAD diagnostic criteria [8]. Nevertheless, it is challenging to quantitatively evaluate behavioral features such as apathy, anxiety, depression, disinhibition, poor insight into difficulties, and impaired decision-making (e.g., poor financial management and impulsivity). Furthermore, patients with these neuropsychiatric features are usually excluded from research studies; therefore, downgrading these manifestations of HIV infection from the current diagnostic criteria may cause a reduction in sensitivity [10].

Further comorbidities that may affect cognition in PLWH include cART neurotoxicity, nutritional and vitamin deficiencies, a history of head trauma or CNS infections, neurodegenerative diseases, trauma at birth, and lifestyle factors such as alcohol or illicit drug abuse.

Although the use of cART has improved the outcomes of HIV infection, it has been suggested that certain cART drugs may worsen the neurological status, with a range of neurological toxicity, from peripheral neuropathy to neuropsychiatric and neurocognitive impairments [111]. Nonetheless, it is challenging to distinguish the specific side effects caused by cART from the direct and indirect impact of HIV itself [112].

Recent research regarding Efavirenz has indicated the presence of toxic side effects, including a reduction in neurocognitive functions. In 2011, Ciccarelli et al. demonstrated that otherwise asymptomatic individuals on Efavirenz exhibited a significantly higher frequency of cognitive impairments than the ‘non-Efavirenz’ PLWH [113]. Further research reported that Efavirenz could lead to an increased risk of toxicity in some individuals, probably due to mitochondrial toxicity [114].

Differentiating the toxicity between the cART at higher concentrations, the most toxic was found to be Efavirenz, Atazanavir, Nevirapine, Abacavir, and Etravirine. Drug regimens, including Darunavir, Tenofovir, Maraviroc, and Emtricitabine, exhibited low toxicity levels with no additive effects with medication combinations [21]. However, more research is needed in this area. Some authors assume that greater care is required when administering cART to balance its positive therapeutic results and potential neurotoxic effects [115]. Nevertheless, other researchers attribute neurotoxicity primarily to viral load and not antiretroviral regimens [116].

A comprehensive presentation on the neurotoxic effects of cART can be found elsewhere [111,117]. 

Any of these comorbidities increase the risk of the misclassification of HAD. The pathology of HIV infection and the associated diseases should be regarded as distinct, overlapping entities. The comorbidities should be recorded as specifiers (e.g., cerebrovascular disease, psychiatric pathology), and if denomination is not possible, they can be labeled as “multifactorial” or due to “undetermined factors” [10].

## 6. Diagnostic Procedures and Workup

In addition to neurocognitive testing, some other ancillary investigations are necessary to diagnose HAD. 

Syphilis serology testing, thyroid studies, electrolyte levels, renal function assessment, and drug screening support the exclusion of other metabolic and infectious etiologies of HAD. Additionally, the levels of vitamin B12 and folic acid should be investigated; if they are borderline, homocysteine and methylmalonic acid levels are more suitable as deficiency indicators.

Among the electrophysiological studies, EEG may help differentiate dementia from epilepsy-associated psycho-organic states, as subclinical seizures may mimic dementia. The EEG is typically normal in early HAD but may demonstrate diffuse slowing without focal abnormalities. Nonetheless, this latter finding is non-specific and may be present in individuals with dementia from any cause, including metabolic causes. Consequently, EEG does not aid the etiologic diagnosis.

The CSF analysis is generally used to diagnose opportunistic infections and CNS lymphoma. In HAD, the CSF is usually acellular, but a mononuclear pleocytosis of up to 20 cells/L may be present [118]. The total protein and albumin levels may be mildly elevated due to the disruption of the blood–brain barrier. In addition, oligoclonal bands and an increased IgG index may be found. However, these findings are non-specific and are often present even in asymptomatic HIV stages. Furthermore, if CSF pleocytosis is found a few weeks after cART initiation, it may be caused by an immunological response in the context of immune reconstitution. 

The CSF studies for excluding a CNS infection comprise the analysis of a cryptococcal antigen, Venereal Disease Research Laboratory (VDRL) test, fluorescent treponemal antibody-absorption test, and Cytomegalovirus polymerase chain reaction (PCR) test. PCR studies may also aid in investigating a possible infection with herpes simplex, varicella zoster viruses, and JC virus as an etiologic agent of progressive multifocal leukoencephalopathy (PML). An elevated HIV RNA concentration can also be found in CNS opportunistic infections, limiting the use of the HIV viral load in the CSF to diagnose HAD [119]. Furthermore, CSF HIV RNA levels do not correlate with cognitive dysfunction. 

CSF markers are useful in the early stages of HAD, when the diagnosis may not be straightforward. Neopterin, quinolinic acid, specific cytokines (e.g., tumor necrosis factor-alpha, interleukin 1, interleukin 6), and antibodies to the gp120 HIV viral envelope protein are correlated with HAD severity. However, they are not widely available and are used only in research. In HAD patients, the CSF levels of Ab42 (a cleavage product of the amyloid precursor protein) were reported as decreased, and the protein tau was found to be increased [120,121]. Additionally, the neurofilament light protein (NFL) and neopterin are frequently elevated, correlating with cognitive impairment [122]. 

The diagnosis of HAD, suspected in the initial evaluation, must be exclusively attributed to HIV. Neuroimaging can assist the HAD diagnosis and exclude other neurologic conditions that may explain or mimic dementia, opportunistic infections, and neoplasms. Although there are no imaging findings that are confirmatory or highly specific for HAD, the role of neuroimaging extends beyond the differential diagnosis of infectious or metabolic processes that mimic the disease; the imaging of the CNS can also be helpful in the diagnosis of other dementias unrelated to HIV infection and for the evaluation of the treatment response in [25]. 

The CT or MRI is usually the initial step of the diagnostic approach, which is required to exclude diseases that can mimic HAD. Usually, imaging demonstrates CNS abnormalities that can be broadly classified into four categories: focal lesions with mass effect, focal lesions without mass effect, diffuse global CNS abnormalities, and ventriculitis or meningitis. Brain CT may detect diffuse cortical atrophy, the enlargement of the ventricles, and increased white matter signals in the advanced stages. Sometimes adults may present basal ganglia calcifications, but they are more frequent in children. Neuroimaging may be normal in ANI and MND. 

Brain MRI is a more sensitive imaging tool. Often, it reveals T2 hyperintense lesions at the level of basal ganglia and deep white matter. The lesions are patchy or confluent, do not enhance with gadolinium, and do not present a mass effect, but this is a non-specific finding. White matter abnormalities may also be seen in other conditions, including small vessel disease, diabetes, and hypertension. With the aging of the HIV population, these lesions need to be differentiated from white matter hyperintensities due to cerebral small vessel disease. Furthermore, both may co-exist. Compared to PML, the cortical U-fibers are not affected in HAD. In the latent HIV stage, there may be some degree of cerebral atrophy. With the progression of disease activity, MRI may detect evidence of interstitial fluid accumulation, with hyperintense signals on T2-weighted images that are more pronounced around the ventricles. The presence of focal contrast enhancement or a space-occupying lesion indicates an alternative diagnosis. Nonetheless, the primary role of MRI is to exclude diseases that may mimic HAD. In addition, in patients with HAD, it is highly unlikely that no suggestive changes on MRI are found [22].

Other brain imaging modalities, such as functional MRI studies, MR spectroscopy (MRS), magnetization transfer ratio (MTR), diffusion tensor imaging (DTI), and voxel-based morphometry, are available only in a few centers. Functional MRI studies reveal abnormalities in regional brain activation during cognitive tasks early in the disease, before dementia can be detected by neuropsychological testing [123]. MRS is a functional neuroimaging technique that measures brain metabolites. N-acetyl aspartate (a marker of neuronal metabolism) may be low due to neuronal injury [124]. In the basal ganglia and white matter, as a result of gliosis and inflammatory changes, choline-containing metabolites (markers of glial metabolism) are increased.

Furthermore, metabolite changes in the basal ganglia and frontal white matter are present even in patients with ANI. Glial activation arises during the asymptomatic stages, and additional inflammatory activity in the basal ganglia and neuronal injury in the white matter corresponds to the development of cognitive impairment. Aging may further exacerbate the inflammation-associated brain metabolites and thereby increase the risk for HAD [125]. Another glial marker, in the myoinositol-to-creatinine ratio (MI/Cr), may be increased at the level of white matter, even from the early stages of cognitive dysfunction [125]. Additionally, the choline-to-creatinine ratio (Cho/Cr) may be increased, and the NA/Cr ratio, as a neuronal marker, may decrease. 

Position emission tomography (PET) detects abnormalities in cortical metabolism. Additionally, it may be helpful in complex cases to exclude CNS lymphoma, which demonstrates increased uptake, whereas the lesions of HAND do not. The single-photon emission computed tomography (SPECT) may show cerebral blood flow abnormalities. Nonetheless, these imaging methods are most valuable for research rather than as standard diagnostic tools. 

Since there is a need for an accurate, early diagnosis of HAD and other milder cognitive impairments, the HAND classification may incorporate the abnormalities or specific neuroimaging features in the future, as they are noninvasive methods of measuring the degree of brain damage caused by HIV infection [22,126,127]. However, despite the progress of advanced neuroimaging methods, there is still much to learn about their application and relevance in HAD patients. Most studies have compared PLWH with HIV-negative controls, focusing on diagnosing HAND or excluding associated comorbidities. Further research is needed to investigate the pathophysiology of the disease in the whole brain, as these investigations may help to predict which HIV-infected patients are at increased risk for HAD and consequently improve the management of PLWH [22].

## 7. Differential Diagnosis of HAD

HAD is a diagnosis of exclusion. The differential diagnosis includes:

- Degenerative diseases: AD, frontotemporal dementia, Parkinson’s disease, Parkinson-plus syndromes, and hydrocephalus.
- Cerebrovascular diseases.
- Toxic-metabolic states (e.g., alcoholism, vitamin B-12 deficiency, thyroid diseases, medication side effects or interactions, and illicit drug use).
- cART that may have adverse effects on neurocognitive functions. For example, depression, insomnia, and impaired neuropsychological testing results were reported after the administration of efavirenz [128].
- Psychiatric diseases, mainly major depressive disorder, can result in cognitive dysfunction (‘‘pseudodementia’’).
- Progressive multifocal leukoencephalopathy (PML).
- Immune reconstitution inflammatory syndrome (IRIS) may be present in PLWH shortly after the initiation of cART therapy. The patients present clinical worsening due to a substantial inflammatory response. The syndrome appears even while the CD4 count improves and the viral load dramatically declines. In addition, IRIS may worsen HAD or PML
- CNS infections, including Creutzfeld–Jakob disease, neurosyphilis, toxoplasmosis, CMV encephalitis, tuberculosis, cryptococcosis, Varicella Zoster virus encephalitis, and other bacterial or viral infections (including SARS-CoV-2).
- Hepatitis-C virus infection.
- CNS lymphoma.

It is essential to keep in mind that most cART-experienced individuals with HAND present a relatively stable level of impairment [118], and any significant changes in cognitive status should be investigated for other causes of deterioration [118] (Table 1).

## 8. Treatment of HAD

To date, the management of HAD and the milder forms of HAND has been based on the best clinical practice, as evidence-based treatments are lacking, with no proven specific intervention for the management of cognitive impairment [133]. Currently, cART is considered the cornerstone of treatment for HAD and HAND, in general, as early treatment with the suppression of viral replication prevents most of the devastating effects of HAD [26]. The administration of cART was reported to lower the virus load in the cerebral parenchyma and the CSF, with improved electrophysiologic parameters and cognitive functions [118,134]. In addition, several studies reported that cART not only had a preventive role for HAND but also induced remissions and decreased the incidence of HAND [118].

Several antiretroviral drugs have a poor CSF-to-plasma drug ratio, suggesting poor CNS penetration. Therefore, a pharmacokinetic and pharmacodynamic scoring system was proposed to indicate the clinical penetration effectiveness (CPE) of different cARTs. The system ranks antiretroviral agents based on their hypothetical and antiviral activity in the CNS [135,136], with Zidovudine, Nevirapine, and Indinavir/r all considered to have the highest CNS penetration. Nonetheless, an evidence base is needed to determine cART for an individual patient based on the CPE score [133]. Based on the results of 23 studies, a recent review concluded that CPE is not as strongly associated with cognitive outcomes as originally hypothesized. However, cART regimens with higher CPE may be associated with modest, improved global cognitive functioning status outcomes and, to a lesser extent, attention/working memory and learning/memory processes [137]. The cART should be administered in line with the greatest evidence-based regimens, as specified by the current guidelines, and based on HIV drug resistance testing [133].

In addition to antiretroviral treatment, the optimal management of other non-infectious comorbidities and depressive conditions is essential in treating PLWH with cognitive impairment. Modifiable risk factors for cognitive dysfunction, such as smoking, substance abuse, sleep disorders (e.g., obstructive sleep apnea), and cerebrovascular risk factors, should be recognized and treated in order to minimize further damage to cognitive functions.

To date, several non-antiretroviral therapies, including minocycline, memantine, selegiline, lithium, valproic acid, lexipafant, nimodipine, rivastigmine, psychostimulants, and others have been investigated for HAND treatment, without significant results [118]. These adjunctive therapies present various mechanisms of action, reflecting the uncertainties of the pathogenic mechanism underlying cognitive impairment in HIV infection [133]. Therefore, the mainstream therapeutic strategies involve actively managing HIV RNA in CSF, modifying cART based on historical and current HIV resistance tests from the plasma and CSF, and assessing and reviewing for toxicities [133].

## 9. Conclusions

Despite recent advances in HIV research, there are many challenges in defining, understanding and treating cognitive impairment in PLWH. Neurocognitive dysfunction is heterogeneous, as either HIV infection or associated comorbidities and concomitant treatments can cause it. Furthermore, as individuals with HIV become older, the prevalence and complexity of cognitive impairment will only increase. The diagnosis of HAD is based on a clinical and neuropsychological examination, with ancillary diagnostic tests that help to exclude other possible causes of neurocognitive dysfunction. Nonetheless, the management of HAD and the milder form of HAND is grounded on best clinical practice, as there is no specific, evidence-based, proven intervention for managing cognitive impairment.

Recent research on evaluating the CNS status, including neuropsychological testing and neuroimaging, focuses on greater reliability in excluding associated diseases and allowing earlier diagnosis. Patients with HIV should be regularly screened for cognitive impairment. Additionally, virologically suppressive cART and the optimization of the confounding conditions and comorbidities are essential aspects of the clinical management of cognitive impairment in PLWH. The early initiation of cART may protect the brain, but further clinical trials on different agents for cognitive impairment are required. 

Research on new emerging infections such as SARS-CoV-2 provides evidence for neurological and cognitive involvement in PLWH. Furthermore, the impact of the COVID-19 pandemic has presented wider consequences for population health than can be attributed to the virus itself. The indirect effects include the increased adoption of telehealth, decreased access to community resources, and social isolation, with a significant health burden; it disproportionately affects older adults and individuals with dementia, that may have limited social networks and increased functional dependence on the community and health system. Teleneuropsychology, including video teleconferencing and telephone interview, is a promising type of neurocognitive assessment providing several benefits, as it permits neuropsychological and other clinical services to continue remotely during situations that restrict person-to-person contact, but further research is warranted in this area. 

## Figures and Tables

**Table 1:** Differential diagnosis of HAD.

Pathologic Condition	Subtype	Diagnostic Procedures
Degenerative diseases	Alzheimer’s dementia Frontotemporal dementia Parkinson’s disease Parkinson-plus syndromes Hydrocephalus	Anamnesis: pattern of clinical signs and symptoms Family history for genetic forms Neurological examination Neuropsychological profile CSF, including biomarkers (e.g., Ab42 and tau) In case of hydrocephalus suspicion, CSF pressure and tap test (drainage of 20–30 mL CSF) Brain MRI
Cerebrovascular disease	Vascular dementia (including multi-infarct dementia, vascular dementia due to a single strategic infarct, vascular dementia caused by lacunar lesions, vascular dementia due to hemorrhagic stroke, Binswanger disease, subcortical vascular dementia, and mixed dementia combined with AD)	Anamnesis: history of stroke Presence of vascular risk factors Neurological examination Brain CT/MRI, angiographical investigations
Toxic-metabolic states	Alcohol abuse	Anamnesis
	Vitamin B-12 deficiency	Plasma vitamin B12, homocysteine, and methylmalonic acid levels
	Thyroid diseases	Thyroid panel
	Medication side effects	Ascertainment of prescription medication
	Drug interactions	
	Illicit drug use	Screening for illicit drugs in plasma and urine
cART	cART with adverse effects on cognitive functions	Medication review (for a review see Yuan et al., 2021 [115])
Epilepsy-associated psycho-organic states	Subclinical seizures that may mimic dementia	EEG is usually normal in early HAD but may demonstrate diffuse slowing without focal findings [129]. Diffuse slowing is non-specific and may be found in individuals with dementia from any cause, including metabolic causes.
Psychiatric diseases	Major depressive disorder (e.g., pseudodementia)	Psychiatric evaluation
HIV specific comorbidities	Progressive multifocal leukoencephalopathy	**PML**: Most PLWH develop PML when the CD4 cell count is <200/µL CSF: usually normal. Proteins may be mildly elevated. Normal CSF findings help exclude other etiologies. CSF pleocytosis may be seen rarely, but the cell count is usually <20/µL. JC virus culture is generally unrevealing. PCR for detecting JC virus has a high specificity (92–99%) and sensitivity (74–93%) [130,131] CT: may demonstrate hypodense lesions MRI: single or multiple confluent lesions, mainly affecting the parieto-occipital white matter, without mass effects. Sometimes infratentorial asymmetrical lesions. The demyelinating lesions include the subcortical U fibers but tend to spare the cortical ribbons and deep gray matter; nonetheless, cases with the involvement of deep gray matter have been reported. If gray matter is involved, there is a scalloped appearance. The spinal cord may be infrequently affected [126]. Brain biopsy: unclear cases. Sensitivity of 74–92% and specificity of 92–100% [131]. Best method to confirm the JC virus in biopsy includes immunohistochemistry or in situ hybridization
	Immune reconstitution inflammatory syndrome	**IRIS**: presentation varies depending on the underlying opportunistic infection or illness. Well known for tuberculosis, PML, cryptococcosis, and toxoplasmosis. In most cases, IRIS occurs within 4–8 weeks of cART initiation or regimen change. A rise in CD4 count often but not always precedes IRIS; it is not a diagnostic criterion. There is no diagnostic test for IRIS; when assessing for possible IRIS, clinicians should exclude HIV disease progression, new infections, opportunistic drug resistance or treatment nonadherence, and drug reactions. MRI: white matter lesions, sometimes with gadolinium enhancement [126]
CNS infections	Creutzfeld–Jakob disease	CSF: 14-3-3-protein and tau EEG: characteristic findings, with periodic or pseudo-periodic paroxysms of sharp waves or spikes on a slow background MRI: hyperintense lesions in the cortical ribbon and basal ganglia, and thalamus on DWI and FLAIR sequences. The “hockey stick” sign, with an increased signal at the putamen and head of the caudate nucleus level (resembles a hockey stick). The “pulvinar” sign, with a bilaterally increased signal at the level of pulvinar thalamic nuclei [132]
	Neurosyphilis	Antibody testing, including CSF analysis. VDRL test, and fluorescent treponemal anti-body-absorption test. MRI: meningovascular syphilis or parenchymatous neurosyphilis
	Toxoplasmosis	CSF: PCR test for T gondii. Elevated protein, variable glucose levels, and white blood cells count. CT/MRI: single or multiple hypodense/hypointense lesions in white matter and basal ganglia, with mass effects and homogeneous or ring pattern contrast enhancement [132]. Neuroimaging may be normal if diffuse toxoplasmosis [126].
	Cytomegalovirus encephalitis	Usually the CD4 lymphocyte counts for <50/µL CSF: PCR confirms CMV with neurologic involvement. Low glucose, elevated proteins, and pleocytosis (neutrophilic or mononuclear) MRI: non-specific. Hyperintense lesions in T2 FLAIR sequences in the periventricular white matter. The presence of enhancement indicates ventriculitis, which can emerge in CMV encephalitis. Mass lesions are very rare. T2-weighted images may demonstrate diffuse white matter hyperintensity (comparable to HIV encephalopathy and other HIV-associated CNS disorders). Gadolinium contrast may indicate meningeal and ependymal enhancement or ring-enhancing lesions [132].
	Tuberculosis	CSF: decreased glucose, elevated proteins, and mild pleocytosis. PCR assays may be diagnostic. Gram staining MRI: leptomeningeal disease, parenchymal abnormalities (e.g., tuberculomas, abscesses, and infarctions), hydrocephalus
	Cryptococcosis	Serum cryptococcal antigen CSF: elevated opening pressure. Normal (25% of cases) and minimally abnormal (50% of cases); therefore, India Ink and serology tests are essential for diagnosis. Culture results are positive for Cryptococcus neoformans CT: non-enhancing, hypodense lesions (cryptococcal pseudocysts). CT findings may be non-specific or normal. MRI: T1-weighted sequences may demonstrate hypodense lesions in the basal ganglia, which are hyperintense on T2-weighted images and may enhance with contrast. Occasionally meningeal enhancement, parenchymal mass lesions without hemorrhage (granulomas), cerebral atrophy, edema, or hydrocephalus [126]
	Varicella Zoster virus	PCR to detect VZV DNA Direct immunofluorescence (DFA) to detect VZV antigen (second choice) CSF analysis MRI
	SARS-CoV-2 infection	In cases of limbic encephalitis, patients may develop anti-N-methyl-D-aspartate (NMDA) receptor encephalitis, autoimmune encephalitis presenting as new-onset refractory status epilepticus (NORSE), steroid-responsive encephalitis, or an unknown type of autoimmune encephaalitis [100]. Autoimmune encephalitis mainly develops several days after respiratory symptoms of COVID-19, suggesting a triggering role of SARS-CoV-2. RT-PCR, CSF analysis, brain MRI
	Other bacterial or viral infections	Blood tests CSF analysis CT/MRI
CNS lymphoma	Non-Hodgkin B-cell lymphoma	Typically CD4 lymphocyte count < 100 cells/mL CSF: elevated protein, pleocytosis; cytology reveals monoclonal malignant-appearing lymphocytes. PCR for EBV DNA confirms the diagnosis of primary CNS lymphoma CT/MRI/PET: most often single lesion, but may be multifocal, mainly located near the ventricles; enhance with contrast; reduced diffusion on diffusion-weighted imaging) The biopsy may be required to differentiate toxoplasmosis and PML lesions from lymphoma [126]. SPECT: thallium-201 (201TI) SPECT scan may help distinguish between lymphoma and toxoplasmosis. Increased 201Tl uptake co-localizing with the lesions on MRI is highly specific for primary CNS lymphoma [126]
Hepatitis—C virus infection	Coinfection	Antibody testing, PCR, and liver function tests

Notes: CSF = cerebrospinal fluid; CT = computed tomography; MRI = magnetic resonance imaging; EEG = electroencephalogram; cART = combination antiretroviral therapy; PML = progressive multifocal leukoencephalopathy; IRIS = immune reconstitution inflammatory syndrome; PLWH = people living with HIV; DWI = diffusion-weighted imaging; FLAIR = fluid attenuated inversion recovery; VDRL = Venereal Disease Research Laboratory; PCR = polymerase chain reaction; CMV = cytomegalovirus; VZV = Varicella-zoster virus; DNA = deoxyribonucleic acid; PET = positron emission tomography; SPECT = single-photon emission computed tomography; SARS-CoV-2 = Severe acute respiratory syndrome coronavirus 2.

## Data Availability

All data are available within the published manuscript.
